# Myelopoiesis and Myeloid Leukaemogenesis in the Zebrafish

**DOI:** 10.1155/2012/358518

**Published:** 2012-07-19

**Authors:** A. Michael Forrester, Jason N. Berman, Elspeth M. Payne

**Affiliations:** ^1^Department of Microbiology and Immunology, Dalhousie University, Halifax, NS, Canada B3H 3J5; ^2^Departments of Pediatrics, Microbiology and Immunology, and Pathology, Dalhousie University, Halifax, NS, Canada B3H 3J5; ^3^IWK Health Centre, Halifax, NS, Canada B3K 6R8; ^4^Department of Haematology, UCL Cancer Institute, School of Life and Medical Sciences, University College London, London WC1E 6BT, UK

## Abstract

Over the past ten years, studies using the zebrafish model have contributed to our understanding of vertebrate haematopoiesis, myelopoiesis, and myeloid leukaemogenesis. Novel insights into the conservation of haematopoietic lineages and improvements in our capacity to identify, isolate, and culture such haematopoietic cells continue to enhance our ability to use this simple organism to address disease biology. Coupled with the strengths of the zebrafish embryo to dissect developmental myelopoiesis and the continually expanding repertoire of models of myeloid malignancies, this versatile organism has established its niche as a valuable tool to address key questions in the field of myelopoiesis and myeloid leukaemogenesis. In this paper, we address the recent advances and future directions in the field of myelopoiesis and leukaemogenesis using the zebrafish system.

## 1. Introduction

The zebrafish is emerging as a powerful model system in which to study haematopoiesis and leukaemogenesis. In addition to the benefits afforded by scale and simplicity of this versatile genetic model system for studying developmental aspects of haematopoiesis, the last decade has seen an explosion of molecular methods and models to facilitate studies informing on haematopoietic disease biology, particularly leukaemogenesis and cancer. At its inception as a cancer model, proliferation and angiogenesis were proposed as phenotypic attributes as readouts relevant to cancer pathogenesis [1]. However, it was the generation of a transgenic zebrafish expressing the *C-myc* oncogene under the control of the *rag2* promoter that went on to develop T-cell acute lymphoblastic leukaemia (ALL), which really revolutionized the view of the scientific world on this small organism as a cancer disease model [2]. In the ensuing 10 years, many models of oncogene induced cancer have been generated in zebrafish along with mutagenesis strategies to identify novel tumour suppressor genes or chromosome instability loci [3–5]. The utility of such models to answer key biological questions continues to grow. In this paper, we focus on developments in the field of myelopoiesis in the zebrafish, cancer models affecting the myeloid lineages, and how these have instructed our knowledge on the biology of these diseases.

## 2. Zebrafish Myeloid Development

Zebrafish haematopoiesis occurs in two waves in the developing embryo, termed primitive and definitive [6]. In contrast to human and murine haematopoiesis (where primitive haematopoiesis initiates with the development of primitive erythroid cells in the blood islands of the yolk sac), in zebrafish, primitive wave erythroid cells develop from caudal lateral plate mesoderm in bilateral stripes that migrate towards the midline forming a structure termed the intermediate cell mass (ICM). A population of primitive macrophages also emerges from a distinct anatomical location in the anterior lateral plate mesoderm (ALPM) between 12 and 24 hours after fertilization (hpf) [7, 8]. Definitive haematopoiesis initiates at around 24 hpf in the posterior blood island (PBI), with the emergence of bipotent erythromyeloid progenitors (EMPs). These cells are marked in their undifferentiated state by combined expression of *gata1 *and *lmo2 *or by expression of* cd41 *[6]. These cells have both proliferative and differentiation potential and increase in number, peaking at 30–36 hpf. This wave of haematopoiesis gives rise to further erythrocytes and myeloid cells and recently has been shown to give rise to early mast cells in developing embryos [9]. Multipotent definitive haematopoietic stem cells (HSCs) expressing *cd41*, *c-myb,* and *runx1* arise directly from *kdrl-*expressing haemogenic endothelium in the ventral wall of the aorta starting around 26–28 hpf [10, 11]. These cells then migrate to the caudal haematopoietic tissue (CHT) where they seed and divide giving rise to all lineages of adult blood cells. These cells go on to populate the adult organs of haematopoiesis in the zebrafish, the kidney and the thymus. The precise timing of the move from primitive wave haematopoiesis to definitive wave haematopoiesis has yet to be fully established, but evidence from globin gene expression and mutants with normal primitive wave blood production suggests that the major contribution of haematopoiesis comes from definitive HSC derived cells by around 5 days post fertilization [12–14].

## 3. Tools for Dissecting Myelopoiesis

Cross-reactive antibodies to zebrafish proteins are lacking, arguably more so in the haematopoietic system than in others. This limitation means that the detailed lineage and differentiation status analysis of haematopoiesis, so elegantly understood in the murine system, is currently challenging to undertake in the zebrafish. Thus a major endeavour in recent years has been the generation of new tools for such analysis in the haematopoietic system. Along with the development of these tools has also come a much broader understanding of myeloid lineage development in zebrafish. The first transgenics developed to mark myeloid cells expressed enhanced green fluorescent protein (eGFP) from the major myeloid transcription factor *pu.1*. Tg(*spi1/pu.1:eGFP*) animals express eGFP in primitive wave myeloid cells but by 2 days postfertilization (dpf), expression of eGFP in myeloid cells is markedly reduced as *pu.1* expression is downregulated [15, 16]. To visualize neutrophil granulocytes later in development, several transgenic lines have been generated by various laboratories. These include the Tg(*lysc:dsRed*) and Tg(*lysc:eGFP*) lines [17] as well as Tg(*mpx:eGFP*) [18, 19] and Tg(*myd88:eGFP*) [20]. While all of these lines label predominantly neutrophil granulocytes, it is notable that the overlap in expression of the endogenous transcripts (by *in situ* hybridization) or protein (by antibody) as well as the reporter gene expression between transgenic lines is not fully concordant, suggesting that subtly different populations are labelled by each transgene depending on the developmental time point of evaluation [17, 21]. Some of these subtleties in gene and protein expression have been addressed. *L-plastin* specifically has in some early studies been suggested to mark monocyte/macrophage lineage cells but there is a clear evidence that this protein is expressed (as in mammals) in all leucocytes [21]. The Tg(*lysc*:*eGFP*) expresses GFP from 22 hpf, initially in primitive macrophages arising from the ALPM. Expression of eGFP increases and is notable in the CHT (likely labelling and differentiating definitive myeloid cells) and the developing brain and retina (more likely to represent the on-going expression in a proportion of macrophages). To clarify precisely which cells express the eGFP from the Tg(*lysc*:*eGFP*) transgene, Hall et al. performed anti-GFP staining along with fluorescent *in situ* hybridization for *mpx, l-plastin*, and *fms. *Dual staining was observed for eGFP with each of these myeloid transcripts; however, there were some eGFP (lysc) expressing cells that did not express *mpx*, some *fms *expressing cells that did not express eGFP (lysc), and some *l-plastin* expressing cells that did not express eGFP. Thus, the Tg(*lysc*:*eGFP*) marks primitive macrophages and a majority of developing granulocytes but does not label all *mpx *positive granulocytes or all* fms* expressing macrophages [17]. It is conceivable that these subtleties may in time come to give us more detailed information about subpopulation of myeloid cells, such as their stage of differentiation. More recently transgenic lines using the *mpeg1* or *fms* (*csf1r*) promoter [22, 23] have been used to distinguish macrophage populations from granulocytic myeloid cells, further enhancing studies of innate immune system. However, *fms* reporter animals exhibit expression in neural crest-derived xanthophores as well as macrophages, which may result in some limitations in the use of this system. By contrast, the *mpeg1* promoter appears exclusive to macrophages, but expression in adult fish is maintained only in zebrafish lines generated using direct transgenic approaches, and not detectable in those lines in which *mpeg1* is linked to a *GAL4/UAS* expression system. To further delineate the expression pattern of macrophages and other mononuclear phagocytes in adult zebrafish, a promoter fragment of the MHC class II beta gene, *mhc2dab, *was isolated. By virtue of the combined transgene expression, the Tg(*mhc2dab:eGFP*) transgenic line in combination with Tg(*CD45:dsRed*) (which labels all leukocytes except B cells) has now allowed identification of macrophages and dendritic cells as well as B lymphocytes in adult zebrafish tissues [24].

**Figure 1:**
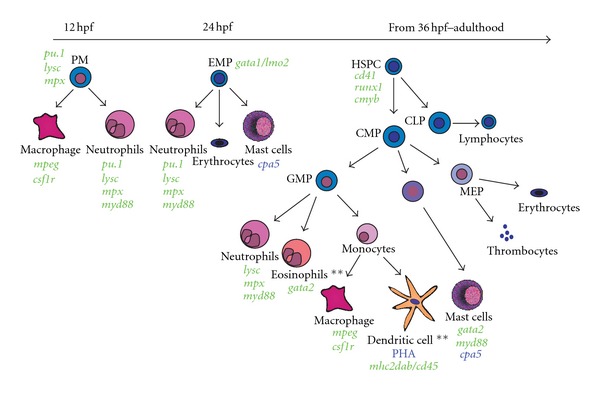
Overview of zebrafish developmental myelopoiesis, key transgenic lines, and lineage identification tools labelling myeloid cell populations during developmental haematopoiesis. (Transgenic lines are shown in green, other specific lineage identifiers are in blue.) PM: primitive myelopoiesis; EMP: erythromyeloid progenitors; HSPCs: haematopoietic stem and progenitor cells; CMP: common myeloid progenitor; CLP: common lymphoid progenitor; MEP: megakaryocyte/erythroid progenitor; GMP: granulocyte/monocyte progenitor; PHA: peanut haemaglutinin. **Denotes lineages only demonstrated in adult zebrafish. Lineage intermediates are shown for clarity but are yet to be isolated as distinct populations in zebrafish.

Several recent studies have also delineated additional granulocytic subpopulations. Zebrafish mast cells can be identified by expression of the *cpa5* transcript, and, like their mammalian counterpart are positive for toluidine blue, express mast cell tryptase and Cd117 at the protein level [25], as well as elements of the Tol-like receptor (TLR) pathway as evidenced by coexpression of *cpa5* and *GFP* in the Tg(*myd88:eGFP*) transgenic line [26]. These cells have also been isolated after fixation by flow cytometry of fast red stained *in situ* hybridization for *cpa5* [27]. The distinction of zebrafish mast cells from zebrafish eosinophils has also been addressed using a BAC-engineered transgenic line expressing GFP from the *gata2 *promoter. This study confirmed the presence of and described in detail the characteristics of zebrafish eosinophils. In the Tg(*gata2*:*eGFP*) line, eosinophils express high levels of eGFP and have high forward and side scatter characteristics by flow cytometry. These cells were also demonstrated to be functionally orthologous to human eosinophils [28]. A summary of transgenic lines and markers facilitating myeloid populations is shown in Figure 1.

As well as facilitating assessment of the ontogeny and spectrum of zebrafish haematopoietic and immune systems, the utility of this array of transgenic animals extends to a more functional analysis of zebrafish haematopoiesis, which will be particularly useful in zebrafish disease models. Once again utilizing cell sorting by flow cytometry, Stachura et al. have established an assay system in which to assess the clonogenic myeloerythroid capability of subpopulations of haematopoietic cells [29]. This recent study utilized traditional clonogenic techniques, commonly used for mammalian haematopoietic cell analysis in methylcellulose, facilitated by recombinant zebrafish growth factors, erythropoietin and granulocyte colony stimulating factor and serum derived from carp. Such studies are in their infancy in the zebrafish system but should lead the way to further capability to assess clonogenic and lineage potential of individual cells and populations. Critically, this will allow more detailed biological analysis of haematopoietic populations which are currently lacking.

## 4. Studies of Developmental Myelopoiesis

Many aspects of myelopoiesis have been interrogated using the zebrafish embryo. Foremost, forward genetic screens have been employed to identify novel genes required for primitive or definitive myelopoiesis. The critical role of transcription factors and developmental microenvironment in determining haematopoietic lineage fate choice has also been elegantly addressed using this model, using reverse genetics and transplantation techniques. More recently transient heterologous overexpression of mutated human oncogenes has provided some mechanistic insight into the potential pathogenetic effects of such genes on normal developmental haematopoiesis and malignant transformation. In addition functional studies have also addressed aspects of the innate immune system using the zebrafish (also reviewed elsewhere in this issue of AIH). What follows is a summary of a selection of studies in zebrafish that highlight its diverse and unique capacity to answer a range of biological questions pertaining to myelopoiesis.

### 4.1. A Myeloid Mutant Identified in a Forward Genetic Screen

Several zebrafish studies have identified novel genes involved in myelopoiesis. Bolli et al. identified the *grechetto* mutant with a mutation within the *cpsf1* gene from an early pressure genetic screen for genes involved in definitive myelopoiesis at 5 dpf. On further investigation, *grechetto *mutants displayed pan-haematopoietic defects, arising from apoptotic cell death of developing haematopoietic stem and progenitor cells (HSPCs). The CPSF1 protein is part of a complex of genes required for processing of the 3′UTR and addition of the poly(A) tail on a subset of pre-mRNAs. CPSF1 specifically recognizes a canonical polyadenylation signal within these pre-mRNAs. Bolli et al., showed that in* grechetto* mutants the transcript encoding the snRNP70 lacked a poly(A) tail [13]. This gene was also identified from a screen for abnormal HSC production [30] and is of particular note because of its role in normal pre-mRNA splicing. Since publication of this report in zebrafish, both loss of function and gain of function mutations in several genes required for normal splicing have been identified as contributing to the pathogenesis of human myelodysplastic syndromes (MDS) [31, 32].

### 4.2. Lineage Fate Choice Studies

Studies in zebrafish embryos have also shed light on the lineage fate decisions during developmental haematopoiesis. Elegant studies of Rhodes and Galloway showed the interplay between the major myeloid and erythroid transcription factors *pu.1* and *gata-1*, respectively, in regulating the fate choice between erythropoiesis and myelopoiesis [33, 34]. Building on these studies Monteiro et al. examined the “bloodless” moonshine mutant carrying a truncating mutation in the transcription intermediate factor-1*γ* (*tif*1*γ*) gene. While previous studies had demonstrated a requirement for *tif*1*γ*  in maintenance of primitive erythropoiesis [35], definitive haematopoiesis had not been examined. In this study Monteiro et al. showed that HSPCs are specified and emerge normally from the aorta in moonshine mutants. Subsequently *tif*1*γ*  is required for normal erythroid differentiation in the CHT at 4 dpf, while expression of differentiated myeloid markers (*mpx* and *l-plastin*) were expanded in the same region. Moonshine mutants also showed increased levels of *pu.1* and reduced levels of* gata-1 *at this time in the CHT suggesting that *tif*1*γ*  may interplay with these transcription factors in the regulation of myeloid versus erythroid fate in progenitor cells derived from definitive HSCs. To determine whether these findings may also be relevant to other stages of haematopoietic development, expression of erythroid and myeloid lineage markers were assessed in moonshine mutants along with *gata-1* and *pu.1* morphants at various time points during developmental haematopoiesis [36]. The authors concluded that *tif*1*γ*  modulates the erythro-myeloid fate choice by regulating the expression of *gata-1* and *pu.1,* and this regulation showed distinct patterns during specific phases of developmental haematopoiesis. This study demonstrated a novel role for *tif*1*γ*  as a regulator of cell fate decisions, and also highlighted the dynamic changes in levels of transcription factors and their interactions that occur during developmental haematopoiesis.

A recent study by Li et al., has also addresses lineage fate decisions between the macrophage versus the granulocytic lineages. In this study the interferon regulatory factor 8 (*irf8*) was identified as a novel regulator of terminal myeloid differentiation downstream of *pu.1*, that promoted the development of the macrophage lineage at the expense of neutrophils during primitive and definitive haematopoiesis [37]. Morpholino knockdown of *irf8* depleted the number of embryonic macrophages and expanded the neutrophil population with the underlying mechanism determined to be a cellular fate switch. There was no definitive evidence for decreased neutrophil apoptosis or increased proliferation to account for increased neutrophil numbers and double-labelling of *l-plastin* and *mpx* or *fms* in *irf8* morphants revealed a predominance of *l-plastin* and *mpx* positive cells. Transgenic overexpression of *irf8* achieved through generation of a Tg(*hsp70:irf8myc*) transgenic line, promoted macrophage development at the expense of neutrophils [37], but could not rescue macrophage development following *pu.1*-morpholino injection. Interestingly, *Irf8*-mutant mice develop a chronic-myelogenous-leukaemia- (CML-) like syndrome with elevated numbers of neutrophils [38, 39]. Taken in this context, this study not only identifies a novel role for *irf8* in normal myelopoiesis, but also highlights mechanisms that could be possibly hijacked during leukaemogenesis.

### 4.3. Functional Assessment of Human Leukaemia Mutations Using Developmental Myelopoiesis

Novel insights into the biology of haematopoietic malignancies have also been gained using zebrafish models expressing haematopoietic oncogenes as detailed in the subsequent section. However, one recent study has harnessed the developmental myeloid phenotype of a zebrafish mutant to functionally interrogate the effects of human nonsynonymous sequence variants (NSVs) found in human acute myeloid leukaemia (AML). In this study *ddx18 *mutant zebrafish were shown to have aberrant myelopoiesis resulting from p53-dependent cell cycle arrest. Sanger sequencing of the *DDX18* gene then identified 4 NSVs in samples from patients with AML. Rescue experiments were then performed using the *ddx18* mutant zebrafish and identified that one of the NSVs appeared to exert a dominant negative effect on developmental myelopoiesis [40]. While this study was based on Sanger sequencing targeting the *DDX18* gene, it paves the way to utilize the zebrafish for other such strategies to interrogate novel NSVs now being identified in the thousands from whole genome and whole exome sequencing efforts, for functional relevance. Furthermore the value of this strategy will become even more powerful as additional models of existing known leukaemic variants and oncogenes become more prevalent, facilitating combined knockdown/overexpression studies using the existing models to test NSVs.

### 4.4. Heterologous Overexpression Studies

Overexpression and knockdown studies of myeloid oncogenes and tumour suppressor genes, respectively, have also been informative in studies using the zebrafish embryo. The nucleophosmin 1 (*NPM1*) gene encoding the ubiquitous nucleolar phosphoprotein nucleophosmin is lost in over one-third of patients with AML or MDS associated with loss of chromosome 5q [41]. In addition heterozygous gain-of-function mutations in *NPM1 *are the most common mutations found in AML accounting for one-third of cases with normal karyotype [42]. Structurally, these mutations result in the generation of a novel nuclear export signal and loss of nucleolar localization signal and thus, in contrast to the normal exclusively nucleolar localization of NPM, mutated NPM is located in the nucleolus, nucleoplasm, and cytoplasm [43]. Furthermore, because NPM contains an oligomerisation domain, NPM mutants relocate at least some of the residual wild-type NPM to the cytoplasm and nucleoplasm. Such NPM mutants have therefore been named NPMc+ to denote their cytoplasmic localization. Heterologous overexpression of the most common *NPM1 *mutation resulting in NPMc+ (NPM mutant A) was undertaken in a study by Bolli et al. Overexpression of NPMc+ resulted in mislocalization of the zebrafish orthologues of *NPM1* (*npm1a *and *npm1b*) to the cytoplasm indicating that human NPM can oligomerize with the zebrafish Npm genes. In addition, primitive myeloid cell numbers were increased, as were *c-myb* expressing cells in the ventral wall of the aorta and *gata1/lmo2* double expressing cells in the CHT. This data suggested that NPMc+ mutant protein led to the expansion of HSPCs as well as developing primitive myeloid and erytho-myeloid progenitor cells [44]. Interestingly, such expansion of myeloid progenitors has also subsequently been demonstrated in a mouse knockin model of NPMc+ mediated leukaemia [45].

### 4.5. Innate Immune System

Cells of the myeloid lineage form the principle components of the innate immune system and, as such, production and development of such cells are stimulated upon exposure to pathogens. G-CSF/CSF3 and its receptor, CSF3R, have well-established roles in haematopoiesis, directing myeloid differentiation of HSCs and proliferation of progenitors [46]. In particular, CSF3 is strongly expressed in response to microbiological toxins in the blood, such as bacterial lipopolysaccharide (LPS), to promote myelopoiesis (especially granulocytes) and cellular migration towards the infection site [47]. Zebrafish possess a homologous csf3/csf3r signalling axis that functions similarly to its mammalian counterpart [48]. Overexpression of *csf3* mRNA expands embryonic myelopoiesis, but loss of zebrafish *csf3r* blocks myelopoiesis entirely with losses of *fms-*, *lyz-*, and *mpx*-expressing populations. Furthermore, exposing embryos to LPS stimulates *csf3* and *csf3r* expression, and leads to an “emergency” increase in *lyz*-expressing granulocytes in a *csf3r*-dependent manner.

Inducible nitric oxide (iNOS/NOS2) signalling also participates in the inflammatory response to infection. The zebrafish homologue, *nos2a*, appears to be dispensable for normal formation of HSPCs [49]. However, using morpholinos and L-NAME or L-NMMA (pan-NOS pharmacologic inhibitors), Hall et al. determined that Nos2a protein is required downstream of C/ebp*β* to expand the HSPC population (as evidenced by increased *c-myb* and *runx1* expression) and promote myeloid differentiation in response to *Salmonella *infection [50]. In this study, zebrafish *nos2a* appears to primarily favour production of neutrophil granulocytes (evidenced by increased *lyz* expression). Hall et al. further confirmed the importance of *csf3r* signalling for “emergency” myelopoiesis during infection, as *csf3r* morphants could not mount a myeloid response upon exposure to *Salmonella*.

## 5. Lessons from Transgenic Zebrafish Models ****of Myeloid Malignancies

Aged wild-type zebrafish (24+ months) are susceptible to the development of a spectrum of neoplasms with an incidence rate around 11% [3], however the incidence of haematopoietic malignancies is rare. Studies of transgenic zebrafish, with tissue specific or ubiquitous promoters driving human or murine oncogenes, have however resulted in faithful models of myeloid leukaemias with features of their human disease counterparts. Below is a summary of the existing models of myeloid leukaemia, the novel findings such models have contributed to our understanding of human myeloid malignancies and a critique of existing and emerging technologies within this field.

### 5.1. K-RAS

Le et al. developed a model of K-RAS-mediated malignant disease by generating a Cre/*lox*-inducible *K*-*RAS* ^*G*12*D*^ allele driven by the *β*-actin promoter. Tg(*β-actin:loxP-eGFP-loxP*:*K*-*RAS* ^*G*12*D*^) zebrafish crossed to a zebrafish carrying a heat shock promoter (*hsp70*) driving *cre* expression resulted in the development of a myeloproliferative neoplasm (MPN) between 34 to 66 days of life, with increased myelomonocytes and myeloid precursors in kidney marrow, and a significant loss of mature erythrocytes [51]. Notably these malignancies occurred in the absence of any heat shock and were rare in animals that had been exposed to heat shock. Sibling animals exposed to heat shock developed more aggressive, nonhaematopoietic neoplasms such as rhabdomyosarcoma and died as a result of these in early life, suggesting that only low doses of activated K-RAS were necessary to transform haematopoietic cells, or that expression of *cre* from the *hsp70 *promoter in the haematopoietic lineage was greater or more leaky than in other tissues.

### 5.2. MOZ-TIF2

Using the *pu.1* promoter to drive transgene expression in myeloid cells, Tg(*pu.1:MOZ-TIF2-eGFP*) fish were the first to demonstrate overt AML in zebrafish at 14 to 26 months of life, showing an accumulation of immature myelomonocytes in the kidney marrow and a reduction in haematopoietic cells within the spleen [52]. It is notable, however, that both Tg(*β*-*actin:K-RAS*) and Tg(*pu.1:MOZ-TIF2-EGFP*) fish showed a low penetrance of disease, and their underlying molecular mechanisms remain unexplored.

### 5.3. Tel-jak2a

A handful of studies have provided more mechanistic insight into oncogenic activity in zebrafish myelopoiesis. In such a study, Tg(*pu.1:FLAG-tel-jak2a*) fish utilized a fusion oncogene created from the zebrafish orthologues of *TEL *and *JAK2*, rather than use of human cDNA [53]. In embryos, *tel-jak2a* expression leads to an accumulation of large myeloid cells in blood smears, induction of the cell cycle, and a gain in cells expressing the myeloid markers *pu.1* and *l-plastin* at 24 hpf. Interestingly, despite a loss of circulating mature erythrocytes by 48 hpf, Tg(*pu.1:FLAG-tel-jak2a*) fish also showed expanded distribution of erythroid markers *gata1* and *βe3-globin* at 24 hpf and 48 hpf. This is in keeping with other studies of Janus kinase/signal transducer and activator of transcription (JAK/STAT) signalling having wide-ranging effects on haematopoiesis in zebrafish embryos. For example, mutant *chordin *zebrafish that overexpress *jak2a* also show upregulation of both erythroid and myeloid genetic markers [54]. This phenotype in *chordin *mutants could be rescued by injection of *jak2a* morpholino or pharmacological treatment with the Jak2 inhibitor, AG490, and phenocopied in wild-type embryos by injection of constitutively active *jak2a* mRNA. This study also suggested that the likely mechanism for the haematopoietic phenotype was hyperphosphorylation of Stat5 because the injection of zebrafish *stat5 *mRNA carrying a hyperactive H298R/N714F mutation led to increases in erythroid, myeloid, and B cell numbers [55]. Similar findings were observed in a zebrafish model of the myeloproliferative disease, polycythemia vera (PCV), where erythroid dysregulation by *jak*2*a* ^V581F^ mRNA could be rescued by injection of *stat5* morpholino [56]. Despite these promising embryonic findings, however, none of the Tg(*pu.1:FLAG-tel-jak2a*) transgenic embryos survived to adulthood [53].

### 5.4. NUP98-HOXA9

Recently, our group described a myeloid-specific, Cre/*lox*-inducible Tg(*pu.1:NUP98-HOXA9*) fish line that exhibits MPN in 23% of fish between 19 and 23 months of life [57]. Despite evidence of myeloid proliferation and delayed cell maturation in kidney marrow, no animals were identified with overt AML. However, mechanistic insights were gained at the embryonic level. Following DNA-damaging irradiation, Tg(*pu.1:NUP98-HOXA9*) embryos showed increased numbers of cells in G2-M transition compared to controls and absence of a normal apoptotic response, which may result from an upregulation of *bcl2*. Furthermore, embryos showed altered haematopoiesis at 28 hpf, with increased myeloid development marked by *pu.1*, *l-plastin*, and *lysc*, at the expense of erythroid development marked by *gata1*, suggesting that expression of the NUP98-HOXA9 fusion oncoprotein is capable of altering the cell fate and myeloid cell differentiation. These early phenotypes in Tg*(pu.1:NUP98-HOXA9)* embryos highlight a potential mechanism whereby HSPCs carrying this oncogene have increased likelihood of acquiring additional mutations due to their impaired DNA damage response and also carry an aberrant population of less differentiated myeloid cells that may be preferentially targeted and thus may mechanistically account for the predisposition of these fish to develop overt MPNs [57].

### 5.5. AML1-ETO

Expression of the *AML1-ETO* oncogene, driven by the heat shock protein 70 (*hsp70*) promoter also results in disruption of developmental myelopoiesis in zebrafish embryos [58]. In this study, embryos show the appearance of cells with blast-like morphology, as well as upregulation of *pu.1* and downregulation of *gata1* at 20–22 hpf. Interestingly, there was a differential impact on more mature myeloid lineages, with increased granulocytes marked by *mpx*, but decreased numbers of cells expressing *l-plastin*. The transforming mechanism was identified as a downregulation of *scl,* one of the master transcription factors for embryonic haematopoiesis. All phenotypes were rescued by injecting Tg(*hsp70:AML1-ETO*) embryos with either *scl* mRNA or *pu.1* morpholino.

To date, the Tg(*hsp70:AML1-ETO*) line represents the most successful use of zebrafish to study the molecular biology of myeloid leukaemia. Despite the absence of an overt adult disease phenotype, Tg(*hsp70:AML1-ETO*) embryos have been an instrumental research tool in the identification of genetic and chemical modifiers of myeloid oncogenesis. A subset of human AML cases show deletions on chromosome 9q, which are specifically associated with the t(8;21) translocation yielding *AML1-ETO.* The effects of del(9q) result from the loss of two genes, transducin-like enhancer of split 1 (*TLE1*) and *TLE4*, in the Notch signaling pathway. A reverse genetics approach used morpholino knockdown of the zebrafish *TLE *homolog, *groucho3*, in Tg(*hsp70:AML1-ETO*) embryos to show an acceleration of the haematopoietic phenotype, namely the appearance of blast-like cells, the increase in *mpx* expression, and a loss of circulating erythrocytes [59]. In human AML, the AML1-ETO  oncoprotein disrupts epigenetic programming through recruitment of histone deacetylase complexes (HDAC), which can be pharmacologically targeted by HDAC inhibitors such as trichostatin A (TSA). Taking advantage of this phenotype, Yeh and colleagues used the rescue of *gata1* expression by TSA as a proof of principle springboard for a chemical modifier screen with a library of known bioactive compounds [60]. Interestingly, they identified COX2 inhibitors, such as NS-398 and indomethacin, as novel therapeutic agents against AML1-ETO, and subsequently demonstrated the critical importance of COX2-prostaglandin E_2_ signalling through the Wnt/*β*-catenin pathway [61] to the altered haematopoiesis in Tg(*hsp70:AML1-ETO*) fish. This proved to be an important discovery—soon after, this same pathway and therapeutic strategy was identified in a mouse model of *Hoxa9;Meis1*-induced AML [62].

### 5.6. Technical Challenges and Advances

The reason behind the long latency and low penetrance of overt myeloid leukaemia in zebrafish models of this disease may lie in part with the lack of available myeloid-targeted promoters that are active in early blood cells. Even with the success of the *pu.1* promoter used in several studies, endogenous zebrafish *pu.1* expression is downregulated during terminal myeloid differentiation, and has been found to be active in only ~2% of adult haematopoietic kidney marrow cells [16]. This could account for the low incidence of AML in Tg(*pu.1:MOZ-TIF2-eGFP*) fish and the lack of progression to overt AML in Tg(*pu.1:NUP98-HOXA9*) fish. Targeted promoters have also proven troublesome in other models of fish leukaemia. Sabaawy et al. showed that expression of the oncogene *TEL:AML1* from ubiquitous zebrafish *β-actin *and xenopus elongation factor 1 (*Xef1*) promoters but not early lymphoid targeted fish using the *rag2* promoter could produce pre-B (ALL) [63]. Such lessons suggest that the use of promoters that are active earlier in zebrafish blood development may prove more robust at driving leukaemic transformation. However, the use of ubiquitous promoters carry the caveat of off-target effects, as seen in Tg(*β*-*actin*:*K-RASG12D*) fish where MPN was one of a spectrum of disease phenotypes, including rhabdomyosarcoma, intestinal hyperplasia, and malignant peripheral nerve sheath tumours [51].

Potency of the oncogenic signal is another hurdle to successfully modelling leukaemia in fish. For example, Tg(*pu.1:FLAG*-*tel-jak2a*) fish as well as the early models of Tg(*rag2:eGFP-Myc*) fish [2] display such severe abnormalities that animals do not survive to breeding age, and so embryos must be reinjected for every study. Cre/*lox*-inducible strategies can be helpful to establish germline transmission of the oncogene, but historically the most reliable method to control Cre activity was to use the *hsp70* promoter, which is known to have leaky expression [51, 57]. This in turn has also suggested that oncogene dosage is likely to have a direct impact on the penetrance and type of malignancies induced as described above for the Tg(*β-actin:loxP-eGFP-loxP*:*K-RASG12D*) [51]. Direct use of the *hsp70 *promoter to drive oncogene expression has proven fruitful in the study of *AML1-ETO*, but the absence of an adult phenotype may reflect the transience of promoter activity following heat-shock activation. Tamoxifen-inducible Cre recombinase (Cre-ERT2) may allow tighter temporal control of transgene expression [64] and can dramatically improve the leaky expression in Tg(*hsp70:Cre*) animals [65]. Hans et al. show that, even at temperature ranges of 37–42°C, recombination events can be blocked completely in Tg(*hsp70:Cre-ERT2*) animals if tamoxifen is not applied following heat shock.

Other intriguing developments include the generation of zebrafish with mosaic expression of oncogenic transgenes [66, 67] allowing more detailed analysis of the effect on oncoprotein expression in individual cells. In mice, the use of lineage-restricted myeloid promoters, for example,* CathepsinG* [68, 69], *Mrp8* [69, 70], has not limited the success of oncogenic transformation and, in fact, committed myeloid progenitor cells have been identified as the leukaemia-initiating cell (LIC) in many karyotypes of AML [69–73]. In the zebrafish, the use of more lineage-restricted myeloid promoters (i.e.,  *lysc*, *mpx*, *mpeg, fms*) have flourished in the field of leukocyte trafficking [17, 22, 23, 74] so these may ultimately provide alternative tools for future fish models of myeloid leukaemogenesis.

Finally, given that overt AML has been achieved in only one zebrafish model to date suggests that the acquisition of mutations within collaborating proto-oncogenes and/or inactivation of tumour suppressor genes may occur less readily in the short life expectancy of the zebrafish. Alternatively, the acquisition of disease promoting cooperating mutations may be masked by increased genetic redundancy that has resulted from the additional round of gene duplication undergone in the teleost genome. However, the zebrafish is well suited to test specific interactions between collaborating oncogenes due to its high fecundity and thus capacity to generate large number of animals with a range of genotypes, as recently demonstrated in neuroblastoma by Zhu et al. [75]. Transgenic fish harbouring multiple oncogenes have also been a successful strategy for modulating the incidence of zebrafish ALL [76]. Thus future strategies to assess the contribution of collaborating mutations could be targeted at overexpression/knockdown strategies of two, three, or four genes.

Until recently, stable gene knockout studies of tumour suppressor genes have been difficult to achieve in most zebrafish laboratories. While the clinical relevance of such models is apparent from mutant alleles derived from targeting induced local lesions in Genomes (TILLING), such as p53 mutant animals [77–79], targeted, heritable gene knockdown in zebrafish has been a major challenge for the community over the past decade. The last few years have seen a major sea change with the snowballing of technical advances in this regard. Initial reports of zinc finger nuclease- (ZFN-) induced cleavage and repair resulting in gene knockouts from two groups [80, 81] followed shortly by the publication of the oligomerized pool engineering (OPEN) system for *in vitro* identification and validation of potential gene targeting zinc fingers by Keith Joung's laboratory [82, 83] have highlighted the potential to harness this technology even in smaller laboratories. Less than 2 years later, the same groups had further refined their *in vitro* and in silico systems to allow accuracy in identification of target sites using bioinformatics alone [84]. Most recently, evidence has shown that transcriptional activator-like nucleases (TALENs), engineered from DNA binding proteins of the Xanthomonas bacteria function even more faithfully in the zebrafish system to target the enzymatic cleavage component of the FOK1 endonuclease to within a few bases of the desired double stranded DNA break [85, 86]. Of course we continue to avidly anticipate the optimization of homologous recombination methodologies to finally permit conditional knockin models of disease. 

## 6. Using the Zebrafish as a Xenograft Model ****for Myeloid Leukaemia

Overall, compared to the lymphoid tumours, models of myeloid leukaemia are relatively less penetrant with leukaemia rates ranging from 25% [51] to <1% [52]. The generation of novel promoters may facilitate more faithful models of human myeloid disease in zebrafish. In particular, dissection of the zebrafish *runx1* promoters has provided new insights into the regulation of this gene in zebrafish but may also prove to be a better driver of oncogene-induced malignant myeloid disease [87]. One potential complimentary strategy is the recent interest in developing methodologies for xenotransplantation of human or mouse cancer cells into zebrafish and applying this approach to myeloid disease [88]. Tissue culture assays and animal models have been instrumental in determining key molecular pathway in cancer and novel drug development. However, *in vitro* assays lack the critical context of the tumour microenvironment, while mouse xenografts are cost-prohibitive and require extensive engraftment time. By contrast, the use of zebrafish facilitates scalability, where large numbers of rapidly developing, externally fertilized transparent embryos can be used to screen compounds in a high-throughput manner. By using embryos at 48 hpf, xenograft rejection is minimized, by virtue of their lack of an adaptive immune system during the first week of life [89].

**Figure 2:**
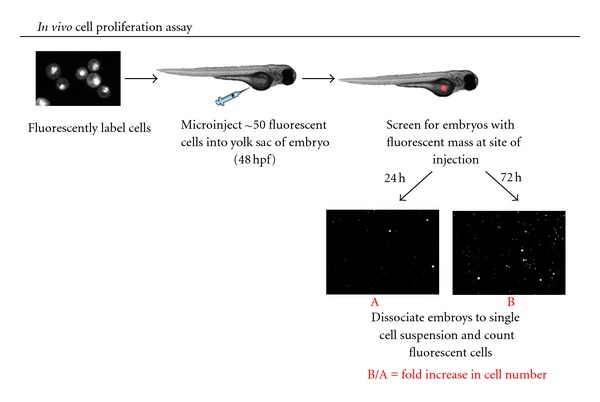
Schematic of *in vivo* cell proliferation assay in xenotransplanted zebrafish embryos. Human leukemia cells are fluorescently labelled with a cell tracking dye. Approximately 25–50 fluorescently labelled cells are microinjected into the yolk sac of 48 hpf casper embryos. Embryos are screened using fluorescent microscopy for the presence of a fluorescent mass at the site of injection. Positive embryos are divided into two groups; one of which is maintained at 35C for 24 h, and the other group is maintained for until the time point of interest with or without drug exposure. At the end of each time point embryos are enzymatically dissociated to a single cell suspension and the number of fluorescent cells in the suspension is counted using a semiautomated macro in Image J (NIH, Bethesda, MD). The number of fluorescent cells present at the later time point divided by the number of fluorescent cells present at 24 h represents the fold increase in cell number. Adapted from Corkery et al. [90].

A number of anatomic sites in the embryo have been trialled for xenografting, but the yolk sac is generally considered the ideal anatomic location and has been used in the leukaemia xenotransplantation studies to date [90, 91]. Incubation of xenografted embryos at 35°C enables growth of injected human cell lines in a fully constituted, 3D, *in vivo* microenvironment, without compromising zebrafish embryogenesis [89, 90, 92]. Two groups, including ours, have exploited xenotransplantation for the study of myeloid leukaemias [90, 93]. Both groups demonstrated successful engraftment and proliferation of CM-DiI fluorescently labelled K562 erythroleukemia and NB4 acute promyelocytic leukaemia (APL) cell lines following yolk sac injection in 48 hpf zebrafish embryos. Moreover, response to targeted therapy with imatinib mesylate in K562 cells harbouring the BCR-ABL1 oncoprotein or with all-*trans* retinoic acid (ATRA), a targeted inhibitor of the PML-RAR*α* oncoprotein found in NB4 cells was observed with the addition of these compounds to the water of xenografted embryos. Pruvot et al. observed a reduction in the number of xenografted K562 cells upon exposure to imatinib and a dose-dependent teratogenic effect and death of NB4 cell xenografted embryos treated with ATRA. Our group have developed a robust *ex vivo* cell proliferation assay to quantify cell numbers over time following xenotransplantation (Figure 2) and demonstrated that xenografted K562 cells specifically responded to imatinib, resulting in decreased cell numbers but no embryonic toxicity. Similar results were obtained with ATRA for xenografted NB4 cells. Importantly, when therapeutic agents were swapped and applied against the opposite cell type, leukaemia cells continued to proliferate demonstrating that human cancer cells can be specifically targeted in a zebrafish xenotransplantation model. These studies open the door for using the zebrafish xenotransplantation platform to rapidly assess the efficacy of novel compounds on the proliferation of human leukaemia cells *in vivo*. Xenotransplantation could also enable screens of currently available anticancer agents for off-label, *in vivo* activity against human leukaemia cells. More recently, as has been demonstrated for some gastrointestinal tumours [94], we have undertaken studies using primary leukaemia patient-derived bone marrow (Tugce Balci, Dale Corkery, Graham Dellaire and Jason Berman, unpublished results). We have seen similar robust engraftment, proliferation, and circulation of primary leukaemia samples and confirmed this process to be an active process, requiring functional living cells, as fixed control cells remained in the yolk. Other groups have further demonstrated differential engraftment of human leukemia subpopulations, with engraftment of CD34+ putative leukaemia stem cells but not from CD34− cells, indicating that zebrafish models may reflect the biology of disease in a similar way as mouse models and enable studies on tumorigenicity and tumour stem cells [93, 95, 96]. In parallel, with other tools, such as the development of syngeneic fish lines (CG1) [76] and the *casper *mutant fish line that permanently maintains transparency into adulthood [97], xenotransplantation will enable the zebrafish to explore questions of leukemia initiating cell frequency, clonogenicity, and the ability to serially transplant disease. Given the complexity of genetic lesions that can present in AML and the heterogeneity of treatment response inherent in this disease, xenotransplantation models could ultimately be used in real-time analysis of primary patient biopsies as an informative diagnostic tool to predict effective therapeutic regimens and/or inform subsequent preclinical murine studies of promising novel agents, ultimately leading to Phase I clinical trials. 

## 7. Conclusions and Future Studies

The zebrafish embryo has contributed significantly to our understanding of the developmental biology of haematopoiesis and myelopoiesis over the past decade. The exponential rise in our ability to dissect the biology of myeloid cells in this small vertebrate will no doubt fuel further insights and broaden the scope for current models of myeloid leukaemias. The advent of TALENs and zinc finger nucleases as well as the zebrafish mutation project at the Sanger Centre (http://www.sanger.ac.uk/Projects/D_rerio/zmp/) promises to deliver us knockouts for all genes in the zebrafish genome that will greatly enhance future studies, particularly of tumour suppressor genes in myeloid disease.

The forward genetic screens that identified so many novel mediators of haematopoiesis in the late 90's [98, 99] including identification of a novel human disease gene [100] have been somewhat out of vogue in recent years. However, completion of the sequencing of the zebrafish genome alongside rapidly reducing costs and improving technology for deep sequencing methodologies are likely to enhance our ability to map such mutations, even in more complex genetic backgrounds. Thus genetic modifier screens of phenotypes observed in myeloid malignancies or development may prove fruitful in the future.

One of the greatest promises for the future of the zebrafish model is its ability to make a significant contribution to the field of myeloid leukaemogenesis by identifying novel therapeutic compounds through chemical screens targeting developmental or early larval phenotypes. The ability to undertake larger scale screening projects even within the environment of academia is becoming more accessible across the zebrafish community and is being enhanced by the application of this platform to xenogeneic cells as well as recent advances in automated image acquisition and analysis capabilities [101]. The growing recognition and acceptance of the zebrafish for studying myeloid biology will enable it to secure a place among other model systems including mouse and cell culture, as a component in a pipeline of preclinical tools to better interrogate molecular pathways and rapidly identify novel therapies with conserved effects across organisms likely to impact outcome for patients with myeloid diseases.
